# A reconfigurable multi-channel on-chip photonic filter for programmable optical frequency division

**DOI:** 10.1515/nanoph-2025-0119

**Published:** 2025-06-24

**Authors:** Simeng Zhu, Bocheng Yuan, Yizhe Fan, Mohanad Al-Rubaiee, Xiao Sun, Zhibo Li, Ahmet Seckin Hezarfen, Anthony E. Kelly, John H. Marsh, Lianping Hou

**Affiliations:** 3526 University of Glasgow, Glasgow G12 8QQ, UK

**Keywords:** microwave photonics, reconfigurable filter, sampled Bragg grating, reconstructed equivalent chirp technology, programmable frequency division

## Abstract

Recent advancements have broadened the application of photon filters based on Bragg gratings within optical communication networks and optical input/output interfaces. Traditional gratings, however, suffer from a fixed refractive index modulation distribution once manufactured, constraining their adaptability and flexibility. This study introduces a reconfigurable multi-channel photon filter on a silicon nitride on insulator platform. The filter incorporates an equivalent linearly chirped four-phase-shifted sampled Bragg grating with micro-heaters to enable thermo-optic tuning, facilitating programmable control over transmission spectral features. Experimental outcomes indicate the filter’s capability to seamlessly transition among single, dual, and quad-band configurations, as well as a band-stop mode, with independent tuning of each band. Moreover, optical frequency division multiplexing experiments using a 50 GHz semiconductor mode-locked laser have affirmed the filter’s tunability. In quad-band mode, band separations of 50, 100, and 150 GHz are achievable; in dual and single-band modes, band intervals extend from 150 to 250 GHz, allowing for precise single-wavelength selection. Featuring high tunability, minimal insertion losses, and superior signal side-mode suppression ratio, this filter structure supports the integration of programmable photonic devices into space optical communications, photonic integrated networks, and elastic optical networks.

## 1 Introduction

Photonics technology has advanced significantly in recent decades, driving the development of high-speed, low-loss, and reliable optical communication networks [1], [2], [3]. Bragg gratings, optical filters that modulate light by refracting periodically, are integral to this progress due to their compact design, low insertion losses, and selective filtering capabilities [4], [5], [6], [7]. These attributes have led to their widespread use in optical communications, photonic integration, and sensor applications [8], [9], [10], [11], [12], [13]. Silicon-based photonic integrated circuits (PICs) have also seen substantial advancements over the last 20 years, with on-chip Bragg gratings playing a pivotal role [14], [15]. These gratings can be integrated with other photonic components to perform complex optical signal processing tasks [16], [17], [18], [19]. However, traditional Bragg gratings are limited by their fixed refractive index modulation, which is predetermined during the fabrication process, hindering their adaptability and dynamic tuning capabilities [20].

Although various methods are available for tuning the spectral response of Bragg gratings, such as temperature control [9], [21], [22], [23] (via either external metal wire offering millisecond-scale response or doped resistive heaters theoretically enabling nanosecond-level response), electro-optic effects [24], [25] (nanosecond-level response with high power consumption), and stress modulation [26], most tuning mechanisms are mainly limited to the shift of the central wavelength. They cannot independently control spectral characteristics such as phase response, band spacing, and bandwidth. However, in key application scenarios such as photonic integrated networks, elastic optical networks (EON) [27], [28], and optical input/output (I/O) interfaces [29], [30], [31], photonic filters (PF) not only need to have tunability of the central wavelength but also support programmable control of the spectral shape to meet the needs for flexible channel allocation and dynamic optical signal processing.

As integrated photonics advances, various emerging materials have been explored to enhance tuning efficiency and response speed [32], [33], including lithium niobate (LiNbO_3_) [25], [34] with strong electro-optic effects, nonvolatile phase-change materials [35] with high stability, III–V semiconductors [36] (e.g., InP, GaAs), and 2D materials like graphene for high-speed modulation [37]. Brillouin photonic materials, such as highly nonlinear fibers and Yb/Er-doped waveguides, have also attracted interest due to their narrowband, high-selectivity filtering properties [38]. Each material platform offers distinct advantages: silicon enables dense passive integration; LiNbO_3_ and III–V materials support efficient electro-optic modulation; graphene provides fast, low-power tunability; and Brillouin materials enable ultra-narrowband, high-Q filtering. In this work, we utilize a silicon nitride-on-insulator (SiNOI) photonic platform, favored for its ultra-low loss, wide transparency window, and CMOS compatibility – ideal for high-performance passive devices, particularly in systems requiring low insertion loss and a high signal-to-noise ratio.

In recent years, with the rapid development of graphics processing unit (GPU) computing power and artificial intelligence technology, the demand for high-throughput, low-latency optical I/O interfaces has continued to grow [39], [40]. Similarly, in data center interconnects (DCI) using GPU, high-performance computing (HPC), and next-generation backbone optical networks, the concept of EON has received widespread attention [41], [42]. Unlike traditional fixed spectral grid wavelength division multiplexing (WDM) systems, EON adopts a flexible and adjustable spectral allocation strategy to improve spectral utilization and reduce network energy consumption. In this context, reconfigurable on-chip PFs have become key devices, used for dynamic optical channel allocation, enhancing the scalability of photonic networks, and improving overall system performance.

Currently, multi-wavelength generation schemes widely used in EON and optical I/O primarily include multi-wavelength laser sources based on distributed feedback (DFB) laser arrays and multi-wavelength multiplexer devices [36], [43], [44], [45], [46]. However, these schemes typically rely on expensive active semiconductor platforms and involve large device volumes and complex manufacturing processes, making it difficult to achieve low-power, on-chip integrated tuneable optical filtering functions. In contrast, reconfigurable optical add-drop multiplexers (ROADM) with their programmable filtering characteristics become an ideal choice for implementing EON [47], [48], [49], [50]. By flexibly controlling the refractive index distribution of the grating, ROADM can dynamically adjust its spectral response, achieving flexible channel multiplexing and optical signal processing.

To overcome these limitations, this paper introduces a multi-channel programmable photonic filter on a silicon nitride-on-insulator (SiNOI) platform. This innovative filter combines two grating couplers with a linearly chirped four-phase-shifted sampled Bragg grating (4PS-SBG) and micro-heaters (MHs). By uniformly introducing four 0.7π phase shifts (PS) within the grating cavity, combined with thermo-optic modulation technology, multiple independently adjustable passbands can be generated in the transmission spectrum. Additionally, optimizing the grating structure with reconstructed equivalent chirp (REC) technology improves phase control precision and enhances manufacturability [51].

Our findings demonstrate that this new filter can seamlessly switch between single, dual, quad-band, and band-stop modes, offering precise control over each passband. This capability is critical for applications in EON and HPC where dynamic spectral management is crucial. Moreover, the proposed filter architecture significantly enhances the signal integrity by reducing crosstalk and improving the overall performance of optical systems. The developed PF not only meets the growing demands for high-throughput and low-latency optical interfaces but also paves the way for more scalable and energy-efficient optical networks. This technology holds great promise for the future of telecommunications and integrated photonic systems.

## 2 Design

PFs play a crucial role in optical communication systems, selectively allowing light of specific wavelengths to pass through for efficient transmission or reflection [52]. To meet high-performance standards, tunable optical filters need a broad tuning range, precise wavelength resolution, and excellent side-mode suppression [53].

Traditional sampled gratings have a coupling coefficient for the +1st order channel that is only about 0.32 times that of a uniform Bragg grating (UBG), resulting in a limited modulation range and lower side-mode suppression compared to UBG. To overcome these limitations, increasing the product of the coupling coefficient (*κ*) and grating length (*L*), noted as *κ·L*, is effective. Enhancing *κ* by deepening the grating etches is one approach, though it raises propagation losses and introduces undesired modes. Alternatively, extending the grating’s length, *L*, compensates for these deficiencies but at the risk of increased scattering losses due to the elongated waveguide structures typical of on-chip sidewall gratings.

This study introduces a novel 4PS-SBG design. This design cleverly divides each sampling period into four segments, introducing a π/2 PS in each, and integrates four 0.7π-PSs across the grating cavity, creating four distinct transmission bands within the +1st order stopband. The stopband’s central frequency can be finely tuned by adjusting the sampling period. The 4PS-SBG significantly boosts the coupling coefficient *κ*, achieving 2.83 times the efficiency of a conventional sampled Bragg grating (C-SBG) without altering the grating’s length *L*. Additionally, it proficiently minimizes interference from 0th and -1st order sub-channels, markedly reducing signal crosstalk and enhancing overall filter performance.

Uniformly inserting four PSs into a sampled grating creates four transmission bands within its +1st order stopband. However, these bands share a resonant cavity due to the uniform grating’s distributed feedback (DFB) characteristics [54], leading to interconnectedness that inhibits independent tuning. This research introduces a linearly equivalent chirped grating that incorporates multiple PSs. This design generates specific reflection points within the grating, enabling light at varying wavelengths near the Bragg wavelength to reflect at distinct grating locations. This setup not only improves phase matching at shifted wavelengths but also minimizes resonant cavity interactions, enhancing the bands’ independent tuning capabilities.

Moreover, the chirped grating supports each band’s photonic isolation, extends the stopbands, and offers a broader modulation bandwidth. Employing REC technology significantly refines the grating’s phase accuracy by two orders of magnitude [55]. This approach surpasses direct modulation of a seed grating in enhancing PS precision, thus ensuring meticulous wavelength control over the filter.

**Figure 1:**
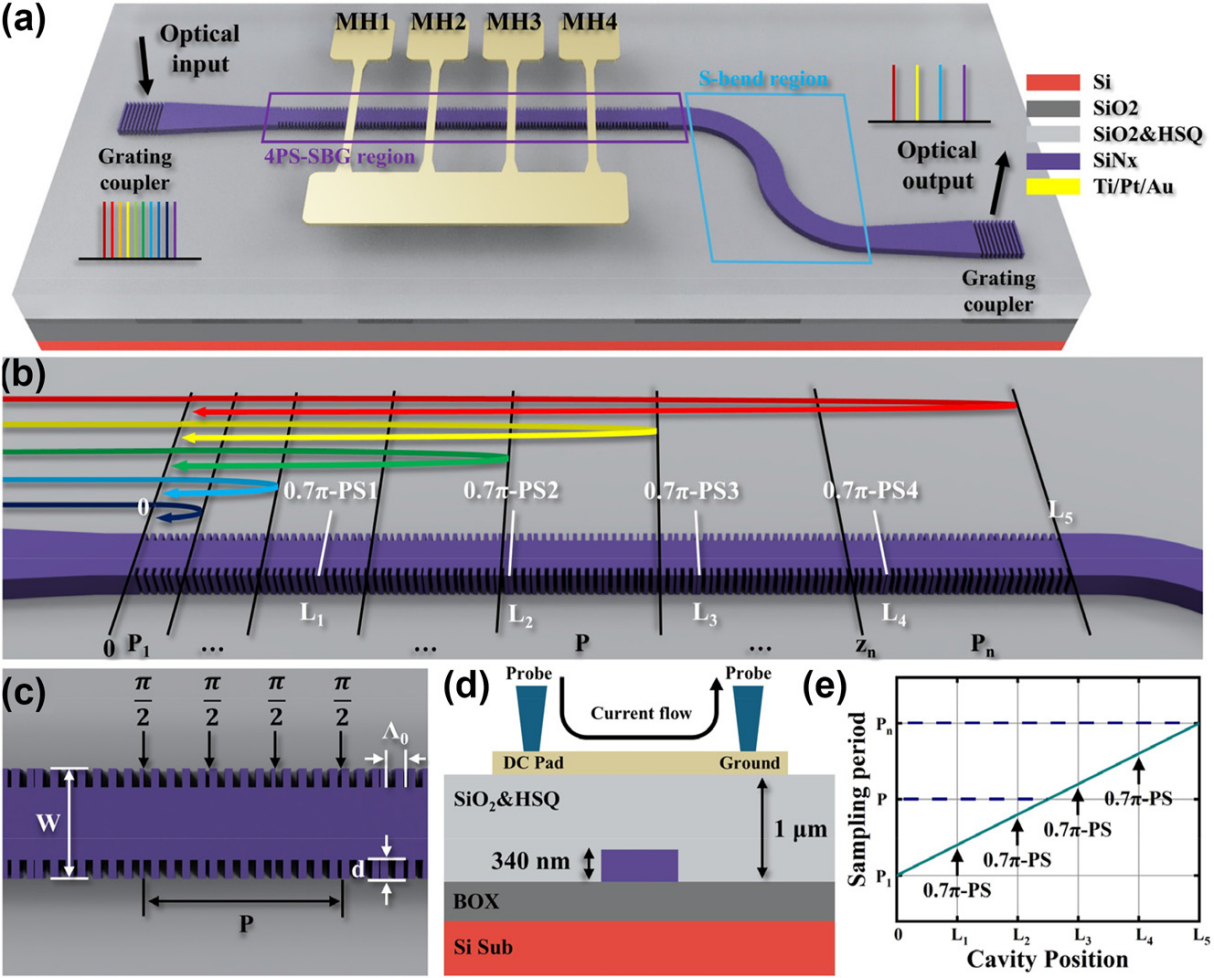
Device schematic structure. (a) Schematic representation of the photonic filter with grating coupler, 4PS-SBG and MHs. (b) Schematic of the grating waveguide described in PF. (c) Enlarged schematic of the 4PS-SBG grating waveguide. (d) Schematic of PF cross-section at MH position. (e) The distribution of the sampling grating period along the whole cavity.

Figure 1(a) illustrates the schematic of the proposed PF, integrated with two grating couplers. The PF comprises a linearly equivalent chirped 4PS-SBG waveguide, an output S-shaped bent waveguide, grating couplers on each side, and four MHs (labelled MH1, MH2, MH3, and MH4, respectively). Positioned above each PS point, these MHs are individually controlled by strategically placed injection electrodes to fine-tune PSs accurately. The MHs link to a common ground electrode, with injection electrodes arranged to ensure electrical isolation and prevent current crosstalk. Applying a direct current to the MH electrodes adjusts the refractive index in targeted areas through the thermo-optic effect, enabling precise local phase shift adjustments. Thermal crosstalk and thermal drift have been shown to be negligible under this geometric design, as confirmed by COMSOL simulations. By strategically programming the current distribution to each MH, the grating’s refractive index modulation distribution can be reconfigured, endowing the PF with diverse spectral properties. It is worth noting that the introduction of the MH induces local refractive index perturbations in the grating units, which may introduce insignificant scattering or mode mismatch. Additionally, evanescent field absorption by the metal can lead to increased loss. However, since the MH covers only a narrow region directly above the waveguide, this effect is considered negligible.

For technical parameters and simulations of MH, please refer to the Supplementary material section S1.

Figure 1(b) illustrates the linearly chirped 4PS-SBG grating incorporating four 0.7π PSs. Conventionally, 1.0π PSs are favored in filter designs for their simpler layouts and effective uniform suppression of side modes within single-channel filters. In contrast, this study employs four 0.7π PSs, which shift the passband blueward and create slightly uneven peak intensities compared with the 1.0π setup. Nonetheless, these shifts adeptly cover the modulation gaps to the left of PB1, allowing for an expanded wavelength modulation range without compromising the side-mode suppression ratio (SMSR). The linear chirp sampling period distribution satisfies the following equation:

(1) $$P_{n}=P_{1}+C\cdot z_{n}$$

where *P*_1_ is the first sampling period, *P*_*n*_ represents the nth sampling period at the start position of *z*_*n*_. *C* refers to the chirp rate, defined as the ratio of the difference between the maximum and minimum sampling periods (*P*_*n*_ − *P*_1_) to the cavity length *L*.

The diagram in Figure 1(b) and (c) details the distribution of sampling periods along the grating’s length. The seed grating operates at a Bragg wavelength of 1,650 nm, with a seed grating period (Λ_*0*_) of 523 nm, a ridge width (*W*) of 1.2 μm, and a recess depth (*d*) of 70 nm. To achieve transmission peaks at 1,547.2 nm, 1,548.4 nm, 1,549.5 nm, and 1,550.7 nm, the grating’s average period (*P*) is set to 7.718 μm, with a chirp rate of 500 nm/mm. At this point, the central wavelength of the +1st sub grating of the equivalently chirped 4PS-SBG is 1,554.5 nm.

Figure 1(d) shows the cross-sectional view of the grating waveguide. MH is manufactured on a 1 μm plasma-enhanced chemical vapor deposition (PECVD) SiO_2_ and hydrogen silsesquioxane (HSQ) cladding surface.

In Figure 1(e), the locations of the 0.7π-PSs within the grating cavity are marked as *L*_1_, *L*_2_, *L*_3_, and *L*_4_, with endpoint designations of 0 and *L*_5_. These are positioned at 190 μm, 380 μm, 570 μm, 760 μm, and 950 μm, respectively.

**Figure 2:**
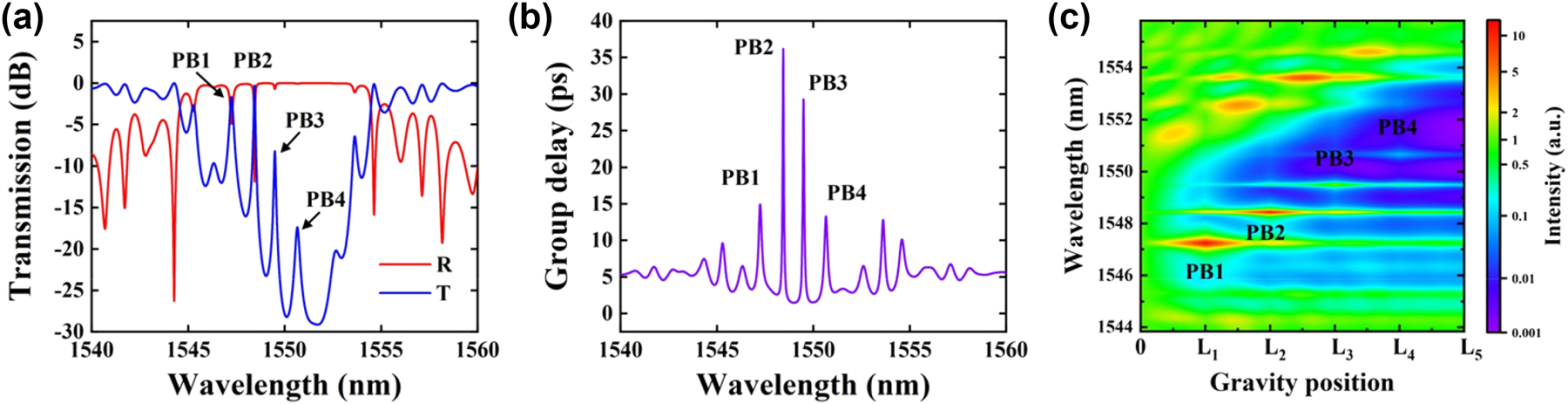
Simulation results of transmission/reflection, group delay, and photon distribution. (a) Simulated transmission (blue line) and reflection (red line) spectra of the PF, (b) time delay spectrum, and (c) photon distribution along the PF cavity, designed using a linearly chirped 4PS-SBG with four 0.7π PSs.

The grating’s optical characteristics, such as its transmission (Figure 2(a)), reflection (Figure 2(a)) and delay spectra (Figure 2(b)), are calculated using the transfer matrix method (TMM) [56]. These calculations reveal four distinct transmission peaks within the stopband, each corresponding to a designated passband – labelled PB1 through PB4 for clarity. Additionally, the photon distribution in Figure 2(c) demonstrates the effective spatial separation of mode fields, significantly reducing resonant cavity cross-interference and ensuring independent and stable operation of each passband.

## 3 Fabrication

This PF was fabricated using a typical CMOS-compatible process at James Watt Nanofabrication Center (JWNC) [19], utilizing 100 kV electron beam lithography (EBL) for precision. Initially, a 340 nm thick SiN_x_ thin film was deposited on the insulator layer using PECVD. This was followed by defining sidewall grating waveguides and grating couplers through a single exposure and inductively coupled plasma (ICP) etching process. Subsequently, the device was coated with a 400 nm thick SiO_2_ thin film via PECVD and a 600 nm thick layer of HSQ resist, which was annealed at 180 °C. This created a 1 μm thick cladding layer that not only prevents the MH metal from directly contacting the waveguides – thus avoiding evanescent field effects – but also enhances surface flatness. This design helps prevent local overheating and melting of heating electrodes due to geometric disparities, extending the lifespan of the heaters. The sample then underwent another exposure, followed by the deposition of a metal layer using electron beam evaporation. The MHs were finalized using a photolithography lift-off technique. The output grating coupler is connected to an S-shaped waveguide with a 100 μm bending radius, effectively minimizing Fabry–Pérot (FP) cavity interference.

**Figure 3:**
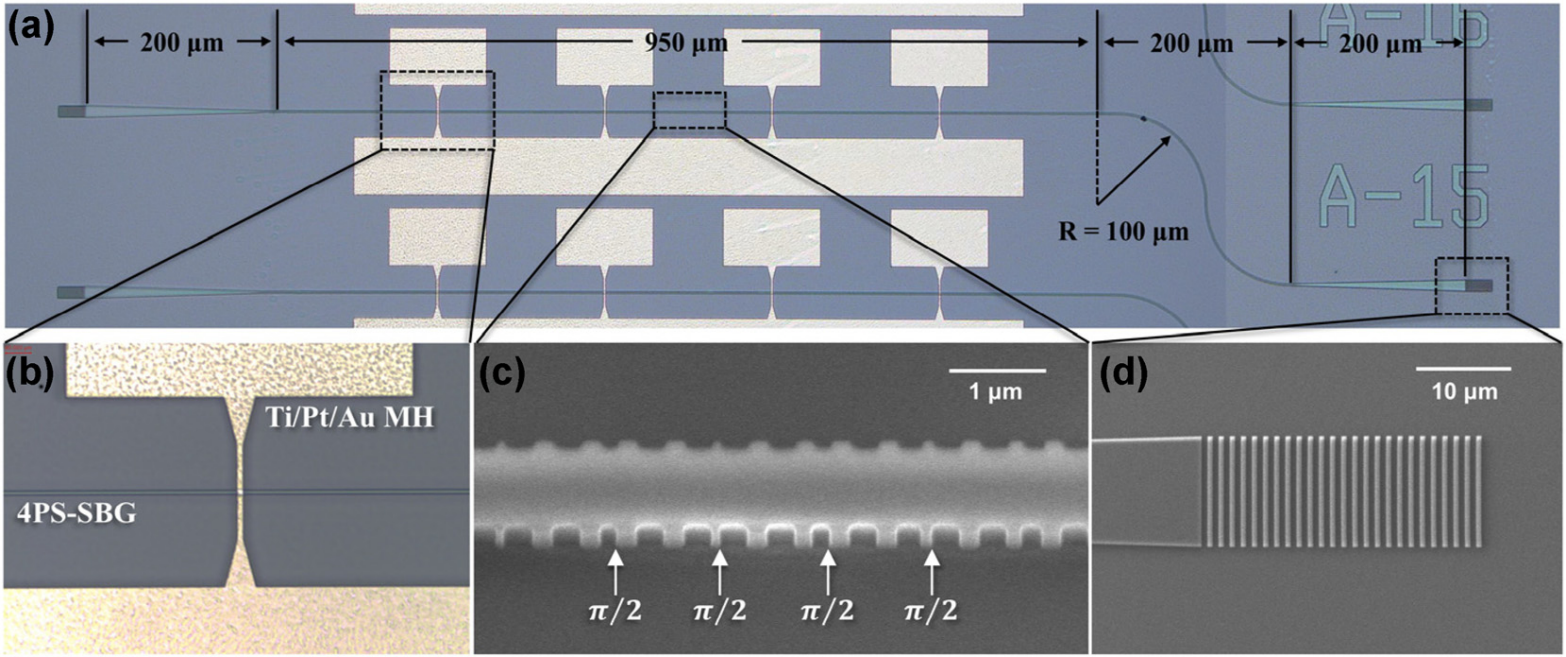
Optical microscope and SEM images of the fabricated device. (a) Optical microscope image of the fabricated device, (b) heating wire of the MH, (c) SEM image of the 4PS-SBG, and (d) SEM image of the output grating coupler.

Figure 3(a) shows a microscopic top view of the proposed PF, measuring 1,600 μm × 300 μm (footprint: 0.48 mm^2^). Figure 3(b) presents a zoomed-in microscope image of the fabricated MH and its relative position to the embedded grating waveguide, while Figure 3(c) showcases the fabricated 4PS-SBG structure under scanning electron microscope (SEM) observation. Additionally, Figure 3(d) illustrates the output grating coupler fabricated on the oxide layer. The MH consists of a top DC injection pad, shared ground pad, and heating resistance wires, with heating wires positioned directly above each PS region. Each wire has a width of 3 μm and a resistance of 27.1 Ω at 20 °C.

For a more detailed description of the fabrication process and the tools used, please refer to the Supplementary material section S2.

## 4 Characterization and results

**Figure 4:**
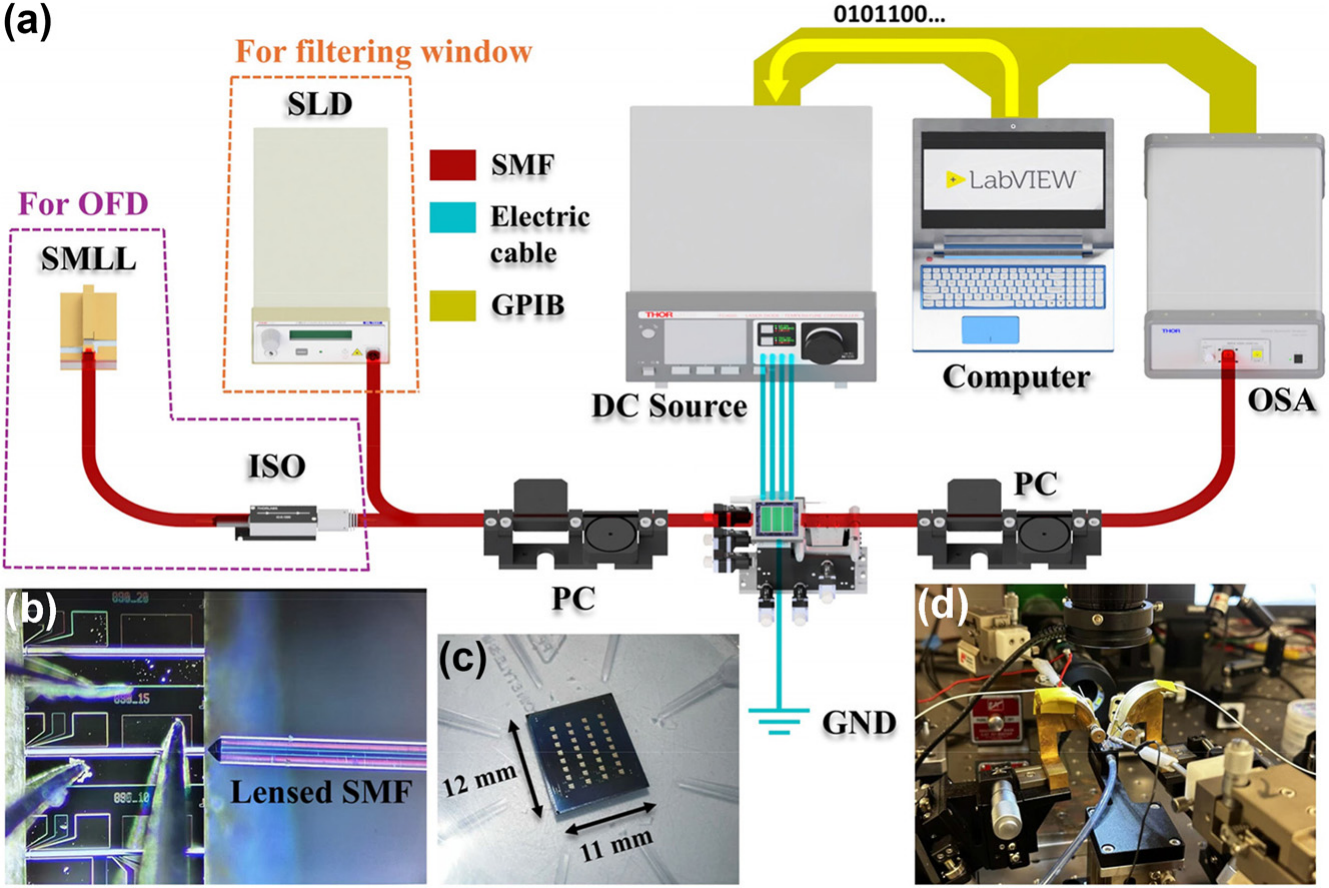
Setup of device characterization. (a) Schematic diagram of characterization setup, (b) light source coupled from the SMLL using a lensed SMF, (c) photograph of the whole chip, and (d) photograph of measurement stage.

**Measurement setup** Figure 4(a) illustrates the experimental setup used for characterizing the device at room temperature. It includes a superluminescent diode (SLD) with a central wavelength of 1,551 nm and a 30 nm −3 dB bandwidth, alongside a 50 GHz semiconductor mode-locked laser (SMLL) centered at 1,550 nm. Single-mode fibers (SMF) connect the SLD to the input grating coupler, with light coupling facilitated by a polarization controller (PC) to ensure transmission in the TE mode. Light from the output is similarly routed through SMF from the GC. Both input and output fibers are aligned directly above the GC with a 15° vertical tilt. The output is analyzed using an optical spectrum analyzer (OSA) with a resolution bandwidth (RBW) of 0.06 nm.

For continuous tuning experiments, only the SLD is utilized, whereas for optical frequency division multiplexing (OFD) applications, the 50 GHz SMLL is the light source (see Figure 4(b)). An optical isolator (ISO) is included when using the SMLL to prevent damage from light reflected off the PF. To assess the filter’s programmable control, different currents are applied to the MH electrodes, affecting resonance peaks in the transmission spectrum, which are measured for shifts. These adjustments allow for rapid, millisecond-scale customization of the grating’s spectral characteristics by independently varying the effective refractive index under each MH [57].

For more detailed technical parameters of the described SLD and 50 GHz SMLL, please refer to the Supplementary material section S3.

See Supplementary material section S4 for grating coupler specific parameters and performance.

The SiNOI sample chip, featuring four columns of reconfigurable PFs with various design structures, is fabricated in a single fabrication run, enhancing efficiency in both fabrication and testing. Figure 4(c) depicts a captured photo of the test chip.

An automated measurement system enhances efficiency, interfacing with instruments via the general purpose interface bus (GPIB) and controlled by LabVIEW software for second level, precise data collection. This setup increases measurement throughput and maintains consistency across multiple experiments, as depicted in Figure 4(d), showcasing the laboratory setup.

**Figure 5:**
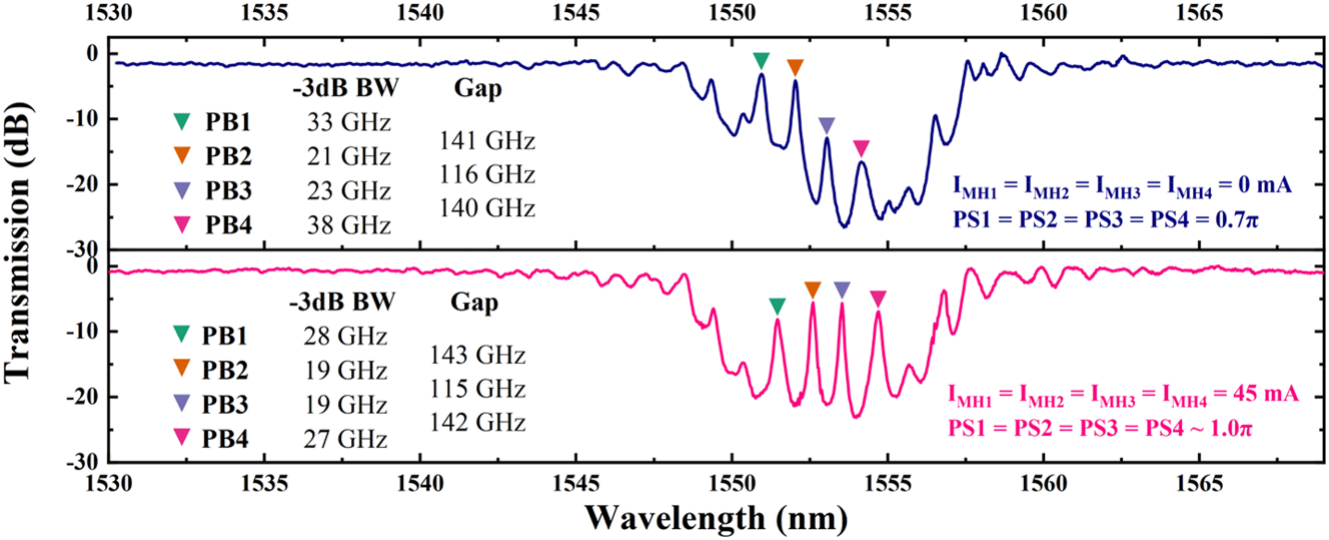
Measured transmission optical spectrum of the PF with all injection currents *I*_*MH*_ set to 0 mA (top blue line) and 45 mA (bottom red line), respectively.

**Filtering window characterization** In this study, we initially analyzed the filtering window and static characteristics of the PF, using an SLD as the light source with the experimental platform maintained at a temperature of 20 °C. The static transmission spectrum was captured and evaluated using an OSA. As shown in the blue solid line in Figure 5, the results highlighted four narrow transmission peaks within the +1st order stopband, typical of multi-phase-shift Bragg gratings. The −3 dB bandwidths for PB1 through PB4 were 33 GHz, 21 GHz, 23 GHz, and 38 GHz, respectively. The frequency spacing between PB1, PB2, PB3, and PB4 was measured to be 141, 116, and 140 GHz. The measured center wavelength of the stopband was 1,553.1 nm, which is 3.6 nm longer than the theoretical prediction. This deviation is likely due to slight under-etching of the SiN_x_ layer (330 nm instead of the planned 340 nm) during the dry etching process. Given that the initial PS was 0.7π instead of the optimal 1.0π, the transmission peaks were not evenly centered within the stopband. Notably, PB1 overlapped with side lobes, leading to uneven peak intensities. By fine-tuning the currents of the MHs, we effectively addressed these intensity variations.

For a vivid demonstration of PF’s programmable spectral features, we initially synchronized the injection currents of all four MHs, aligning the peaks centrally within the stopband, effectively simulating a uniform 1.0π PS, setting each MH’s current to 45 mA (see pink line in Figure 5). The new frequency spacing between PB1, PB2, PB3, and PB4 is measured to be 143, 115, and 142 GHz, and their respective −3 dB bandwidths are 28 GHz, 19 GHz, 19 GHz, and 27 GHz. It is worth noticing that the resulting passband spacing remains almost unchanged during the modulation of the PSs from 0.7π to 1.0π and the passband −3 dB bandwidth is slightly reduced, which means that the four PSs are independent of each other and work stably, and the spectral resolution increases as modulation occurs. At this point, all passbands within the stopband exhibit a characteristic Lorentzian lineshape. The ERs of PB1, PB2, PB3, and PB4 are 15.0 dB, 17.5 dB, 17.4 dB, and 16.1 dB, respectively. As an optimization strategy, ER uniformity and flattening can be achieved by increasing the PS spacing or through inverse design, where ER uniformity is imposed as a constraint to jointly optimize the number, positions, and profiles of the PS elements. However, these approaches typically come at the cost of reduced photonic independence between the passbands.

**Figure 6:**
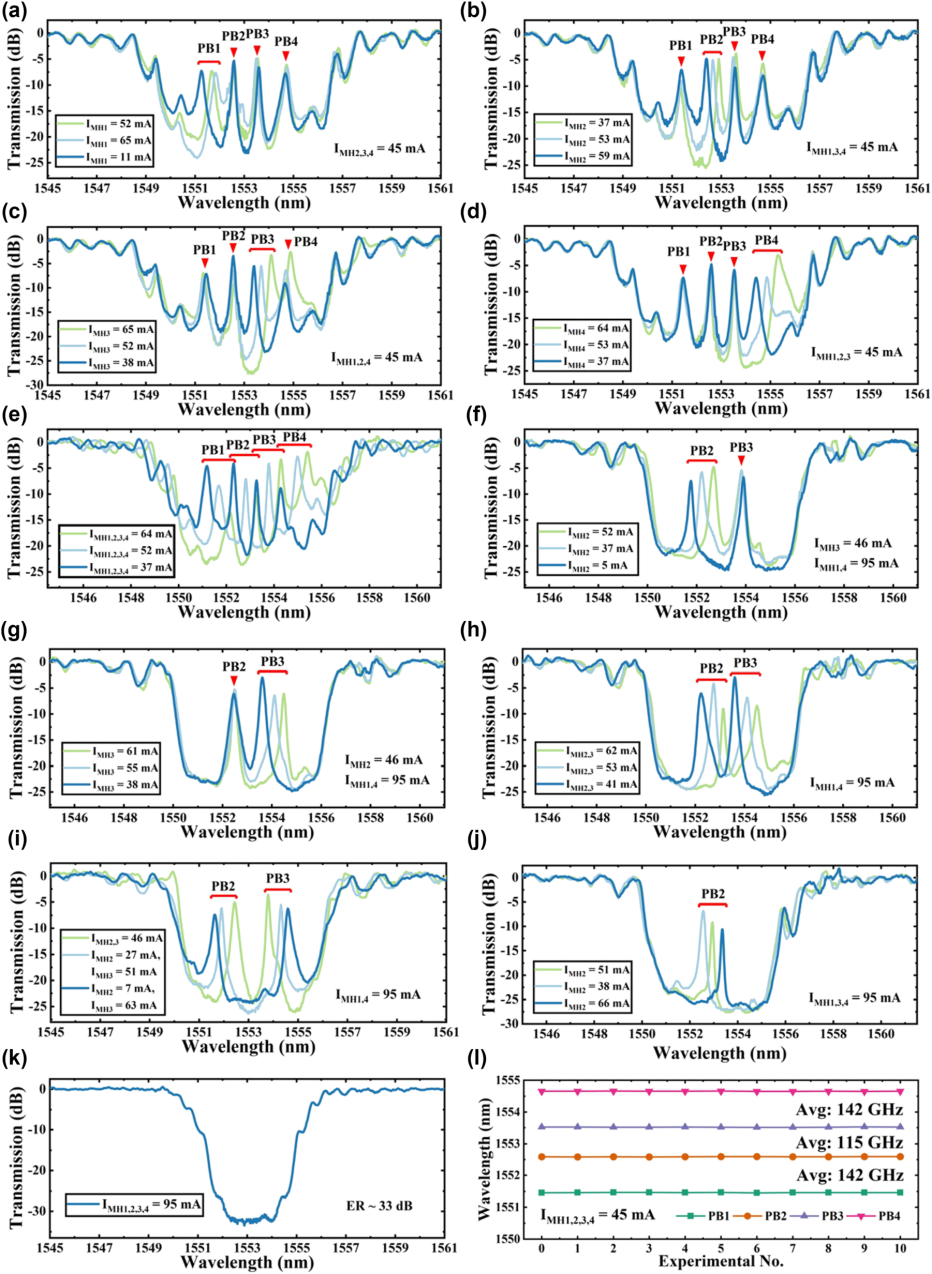
Measured spectra of the fabricated grating filter with an SLD input under different modulation conditions. (a–d) individual modulation of MH1, MH2, MH3, and MH4 in quad-band mode; (e) simultaneous modulation of all MHs in quad-band mode. In dual-band mode: (f–g) separate modulation of MH2 and MH3, (h) simultaneous modulation of all MHs with constant spacing, and (i) simultaneous modulation with variable spacing. Measured spectra for (j) single-band mode with MH2 modulation and (k) stop-band mode. (l) Spectral analysis of center wavelength and spacing stability in 10 repeated simultaneous modulation experiments in quad-band mode.

We then individually adjusted the currents at each PS point to examine their effects on the transmission spectrum (refer to Figure 6). The experiments indicated that adjustments to individual PSs primarily affected their respective transmission peaks, with the overall structure of the +1st order stopband remaining stable. For instance, in Figure 6(a), adjusting only the PS1 current shifted PB1’s center frequency by 72 GHz, without significantly affecting other peaks. The most considerable shift was a 12 GHz adjustment in PB3, substantially below twice the RBW, highlighting effective optical isolation between passbands and confirming the successful independent modulation capability of each passband. Additionally, the stopband width remained essentially constant throughout these adjustments. Similarly, when PS2 was adjusted in isolation (as shown in Figure 6(b)), PB2’s center frequency shifted by 62 GHz, with the largest deviation observed in PB3 at 14 GHz, staying under two times the RBW. Adjusting PS3 (Figure 6(c)) primarily affected the longer wavelengths, resulting in a 90 GHz shift in PB3’s center frequency and a 23 GHz shift in PB4, while the peaks in the shorter wavelength range remained stable. Modulating PS4 (Figure 6(d)) had a significant impact on PB4, shifting its center frequency by 115 GHz, with minimal effect on the shorter wavelength passbands.

The single-band modulation experiments (Figure 6(a)–(d)) demonstrated that the overall structure of the stopband and the extinction ratio (ER) remained constant, only altering the central frequencies of targeted passbands. This underscored the effective optical isolation between the PS points, affirming the filter’s capability for independent adjustment.

Applying thermo-optic modulation simultaneously to all four PS points (Figure 6(e)) resulted in a global frequency shift across all transmission peaks, rather than isolating changes to individual passbands. The frequency intervals between the peaks – PB1 to PB2, PB2 to PB3, and PB3 to PB4 – remained consistent at 143 GHz, 115 GHz, and 142 GHz, respectively. This illustrates that coordinated multi-point modulation can shift the filter’s entire spectrum without altering the relational frequencies between passbands. Furthermore, during the synchronized modulation of all four points (Figure 6(e)), the form of the grating’s stopband was essentially maintained. Only minor redshifts occurred at the stopband edges as the transmission peaks neared these boundaries, slightly broadening the stopband width. These results indicate that the filter not only supports precise adjustments via individual PSs but also accommodates global spectral shifts through collective phase modulation, greatly enhancing its tunability. The above modulation process is defined as the quad-band operating mode of the PF.

The ability to modulate PS amplitude via MHs allows for dynamic selection of the filter’s passbands. Specifically, adjusting the PS amplitude to an integer multiple of 2.0π effectively nullifies it, akin to switching it off. This capability has enabled us to implement both dual-band and single-band filtering modes. For instance, in certain experiments, only PS2 and PS3 are activated, with PS1 and PS4 set to 2.0π. In this configuration, only PB2 and PB3 remain within the stopband of the grating, while PB1 and PB4 experience red shifting due to thermo-optic effects. As PB4 shifts, it gradually merges into the sideband and moves beyond the stopband, while PB1’s intensity decreases during red shifting and eventually fades, becoming submerged within the stopband. This phenomenon is referred to as the dual-band mode conversion.

In our experiments illustrated in Figure 6(f)–(i), we explored the PF of dual-band mode, where only the passbands generated by PS2 and PS3 are active within the grating’s stopband. We estimate that the amplitudes for PS1 and PS4 are set to 2.0π. Initially, as depicted in Figure 6(f), when only PS2 is modulated, PB2 shifts 118 GHz, while the center frequency of PB3 exhibits a minimal fluctuation of 9 GHz, indicating stability. Figure 6(g) presents the response when only PS3 is modulated; here, PB3’s center frequency shifts by 110 GHz, whereas PB2 shifts slightly by 3 GHz, remaining below the OSA’s resolution RBW.

In coordinated modulation experiments shown in Figure 6(h), despite a 112 GHz modulation, the frequency interval between the two active peaks remains constant at 168 GHz. This stability is crucial for the PF’s operational efficiency in dual-band configurations. Figure 6(i) highlights our precise control over the injection currents for PS2 and PS3, achieving a separation of the dual bands’ center frequencies from 168 GHz to 367 GHz while maintaining the central wavelength. This capability to dynamically adjust the distance between peaks underscores the PF’s sophisticated tuning capacity, enabled by effective management of the thermo-optic effect. This functionality demonstrates the PF’s potential in applications requiring precise spectral adjustments and robust filtering capabilities.

In single-band trials (Figure 6(j)), only PS2 is operational, making PB2 the sole transmission peak. The results from these tests indicate that the filter maintains the stopband’s overall structure and exhibits excellent single-channel filtering performance. Additionally, the ability to alternately toggle PS1 through PS4 enables versatile configuration of filtering windows across the entire stopband, allowing for comprehensive spectral adjustments.

When all PSs within the grating are turned off, as illustrated in Figure 6(k), the grating’s spectral response resembles that of a normal grating without any defects. This scenario is characterized primarily by chirping effects that suppress the side lobes in the +1st order stopband, effectively transforming the filter into a band-stop filter. It’s important to note that in this configuration, due to the absence of resonant cavities that would normally accumulate energy, the noise floor of the filter is significantly reduced compared to configurations with active passbands, achieving an ER of 33 dB.

For DWDM applications, out-of-band suppression is also a critical parameter. As shown in Figure 6, the out-of-band suppression varies across different operating modes: in the quad-band mode, it ranges from 20.5 dB to 25 dB; in the dual-band mode, from 23 dB to 26.4 dB; in the single-band mode, it stabilizes around 27.4 dB; and in the stopband-only mode, the maximum suppression reaches 33 dB.

To assess the device’s repeatability, we conducted an experiment, as shown in Figure 6(l), by repeatedly injecting a 45 mA current 10 times in an unmodulated state. We recorded the central wavelengths of PB1, PB2, PB3, and PB4 in each iteration. The results consistently demonstrated that after 10 full modulation cycles, the central wavelengths of each passband remained stable, with no significant variation in frequency intervals. This confirms the device’s excellent repeatability and reliability.

These experiments confirm that by independently or collaboratively modulating multiple PSs, we can flexibly and globally control the filter’s peak positions, peak intervals, and stopband characteristics. The presence of four PS points enhances the filter’s spectral complexity and programmability. Additionally, the results demonstrate that adjusting the PS amplitude can dynamically regulate the number of active passbands, enabling transitions from quad-band to dual-band, single-band, and even band-stop modes. This adaptability expands the filter’s applications in reconfigurable photonic signal processing, optical frequency division multiplexing, and precise spectral modulation.

**Programmable optical frequency division** We further analyzed the modulation characteristics of the transmission spectrum and the variation trends of the filter windows to evaluate the device’s applicability in OFD. The experiment employed an SMLL as the input light source, which has a 50 GHz free spectral range (FSR) and generates a uniformly spaced optical comb output. As the frequency components pass through the modulated PF, the filter’s window settings selectively shape the output spectrum by enabling specific spectral selections or suppressions. By adjusting the modulation states of different PS points, we can precisely control the position and spacing of the laser modes, enabling dynamic reconstruction of the input optical comb spectrum.

**Figure 7:**
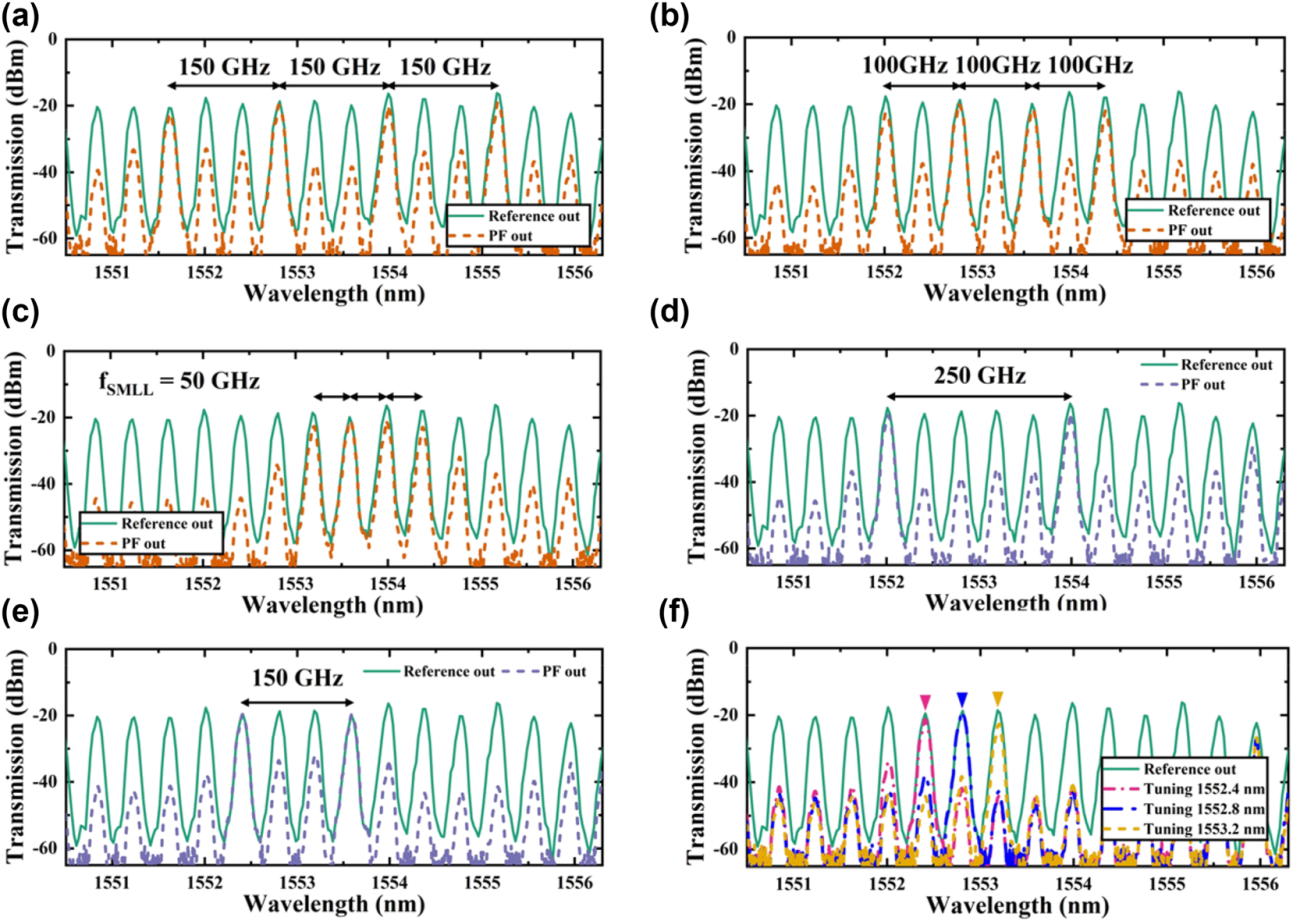
Output spectra of OFC signals from the SMLL and SiNOI ridge waveguides (green solid line), and OFC signals filtered by the PF (dashed line) with a quad-band filtering setup spaced at (a) 150 GHz, (b) 100 GHz, and (c) 50 GHz, and a dual-band filtering setup spaced at (d) 250 GHz, (e) 150 GHz, with settings for selecting individual laser modes (f).

As depicted in Figure 7, the green solid line shows the reference signal post-transit through an equal-length ridge waveguide, while the other curves represent the output spectra post-filtration. In the experiments (shown in Figure 7(a)–(c)), the filter was configured to operate in a four-band mode. In Figure 7(a), the filter enabled transmission at 150 GHz intervals (i.e., triple mode-locking frequency of the SMLL, *f*_*SMLL*_), allowing only every third input SMLL spectral line to transmit while suppressing others, creating an equidistant four-wavelength laser structure. Figure 7(b) shows the filter with a reduced passband interval of 100 GHz, setting the frequency interval between two adjacent selected transmission lines at 100 GHz. Figure 7(c) demonstrates a 50 GHz interval filtering mode, where the intervals of the transmission spectrum perfectly match the FSR of the input source, and unselected optical frequency components remain markedly suppressed. Throughout all the above configurations, the SMSR exceeded 10 dB, satisfying the optical network’s requirements for signal quality. Notably, the minimal insertion loss in the quad-band optical frequency division multiplexing experiment was observed in the 50 GHz filtering mode, with PB2 at 1,553.6 nm showing an insertion loss of only 1.5 dB. Due to the limitation of our *f*_*SMLL*_, the minimum spectral resolution demonstrated here is 50 GHz. However, the theoretical −3 dB resolution can reach as low as 19 GHz. As an optimization approach, employing buried grating structures or multi-phase-shifted (>4) sampled Bragg gratings can effectively enhance the filter resolution.

In our optical dual-frequency division multiplexing experiments (illustrated in Figure 7(d) and (e)), we reduced the number of filter windows to two while maintaining the flexibility to adjust the intervals between passbands. Specifically, in Figure 7(d), we achieved a 150 GHz separation, while in Figure 7(e), we expanded the interval to 250 GHz. These configurations demonstrate the filter’s ability to transmit widely spaced frequency components while effectively suppressing others, even with fewer passbands. In these dual-band configurations, the minimal insertion loss recorded was 0.5 dB for PB3 at 1,553.6 nm, with the SMSR exceeding 15 dB. This performance underscores the filter’s capability to selectively transmit signals over varied intervals while ensuring high signal integrity, making it ideal for precise optical signal processing and RF signal transceiver tasks [58], [59], [60].

In the single-frequency division experiment (shown in Figure 7(f)), we focused on a solitary narrow-band transmission window, tuning laser modes specifically at 1,552.4 nm, 1,552.8 nm, and 1,553.2 nm, each separated by 50 GHz. The transmission spectrum in this setup effectively isolated the target optical frequencies, significantly dampening other modes. This precise control within a narrow-band spectrum suits applications requiring selective channel operation and narrow-band filtering, with the single-band mode recording a minimal insertion loss of 1 dB at 1,552.8 nm and maintaining an SMSR over 15 dB.

Overall, our experiments validate the PF’s ability to dynamically manipulate optical frequency division multiplexing signals by finely tuning multiple PSs, either independently or in coordination. The device adeptly supports a range of operational modes from four-channel to single-channel, employing precise phase adjustments to facilitate flexible and programmable filtering actions. Additionally, our results highlight the PF’s efficacy in maintaining precise spectral control with low insertion loss and high SMSR, proving its significant potential for applications in high-speed optical communications, adaptable photonic signal processing, and optical frequency comb (OFC) technology. As a potential solution, the use of an industrially mature heterogeneously integrated laser approach could be adopted in the future to further optimize insertion loss and related performance issues [61].

It is important to note that our architecture is scalable and can be extended to support more PSs regions. However, too many PSs in a narrow stopband cause merged or broadened passbands. This reduces filtering resolution and transmission intensity, especially near stopband edges. Longer gratings are needed to preserve passband independence. This increases scattering loss and insertion loss due to fabrication limitations. Non-uniform PS spacing can help equalize peak spacing. More PSs also require more MHs, raising power consumption and reducing yield.

## 5 Conclusions

In this research, we presented a reconfigurable multi-channel photonic filter built on a SiNOI platform, utilizing a 4PS-SBG with an equivalent chirp for programmable OFD. This innovative structure enables independent manipulation of multiple transmission passbands within the Bragg stopband, providing versatile spectral control. Experimental results demonstrated that the integrated MHs allow for precise local adjustments in PS regions, offering over 100 GHz of independent tuning for selected passbands, while effectively minimizing fluctuations in unmodulated passbands (below twice the RBW) and maintaining a high SMSR and low insertion loss.

Using a 50 GHz SMLL as the input source, we tested the filter’s capability to dynamically reconstruct OFD channels. Our results showed tunable channel intervals ranging from 50 GHz to 250 GHz, supporting a broad spectrum of optical signal processing applications. Additionally, the filter’s ability to transition between single, dual, and quad-band modes highlights its extensive reconfigurability and functional diversity.

This reconfigurable photonic filter offers a forward-thinking solution for future PICs, leveraging localized thermo-optic tuning for precise spectral programmability. Its robust performance, compact design, and compatibility with CMOS manufacturing enhance its potential, particularly for accurate wavelength selection, dynamic optical signal management, and versatile photonic integrated systems. With significant research and application potential, it is poised to advance deep space network (DSN), dynamic optical signal processing, and adaptable optical networks, making it a valuable asset for next-generation photonic technologies.

## Supplementary Material

Supplementary Material Details
