# Supplementary material for: A reconfigurable multi-channel on-chip photonic filter for programmable optical frequency division
Source: Nanophotonics. 2025 Jun 24;14(15):2619–31. doi: 10.1515/nanoph-2025-0119 (PMC12322728; doi:10.1515/nanoph-2025-0119)
Supplement: Supplementary file 1 — Supplementary Material Details [file j_nanoph-2025-0119_suppl_001.docx]

A Reconfigurable Multi-Channel On-Chip Photonic Filter for Programmable Optical Frequency Division: Supplemental Document

Simeng Zhu,* Bocheng Yuan, Yizhe Fan, Mohanad Al-Rubaiee, Xiao Sun, Zhibo Li, Ahmet Seckin Hezarfen, Anthony E. Kelly, John H. Marsh, and Lianping Hou

James Watt School of Engineering, University of Glasgow, Glasgow G12 8QQ, U.K.

*2635935z@student.gla.ac.uk

This PDF file includes:

Section S1 to S4

Figure S1 to S6

Equation S1

1. Design and characterization of Micro-heater

Typically, thermo-optic phase shifts in waveguides can be facilitated either by integrating MHs directly above the waveguides [1] or by utilizing the resistive properties of doped waveguides [2]. The former approach necessitates a thick protective cladding layer to shield the optical propagation modes from metallic interference, effectively reducing optical losses due to the evanescent field. Although this method might be slower in response compared to using doped waveguides, the integration of metal MHs simplifies the manufacturing process by eliminating the need for ion implantation and annealing processes.

In this study, we have employed metal MHs to achieve the desired thermo-optic phase shifts. Our silicon nitride waveguides have a cross-sectional size of 1.2 μm in width and 0.34 μm in height. The SiN_x_ layer is on a 5 μm thick oxide layer and covered with a 1 μm thick oxide cladding layer. The MHs are constructed from a multi-layered composite of nickel-chromium (NiCr), platinum (Pt), and gold (Au), with a total thickness of 0.23 μm. NiCr acts as the adhesion and initial heating layer due to its high resistivity, while Pt enhances the heating efficiency, and Au provides necessary oxidation and corrosion resistance. The heater wires themselves are 3 μm wide.

After the fabrication process, testing at room temperature revealed that the resistance of a single heating wire stands at 27.1 Ω.

The heating distribution of the PF is simulated using COMSOL Multiphysics in a three-dimensional model (Fig. S1). The relationship between the phase amplitude shifts of the waveguide and the temperature change, *∆T*, is given by [3]:

|  |  | (S1) |
| --- | --- | --- |

Here, *n_eff_* represents the effective refractive index, and *L* is the modulation cavity length. MH1, MH2, MH3, and MH4 are thin-film heaters placed on top of the cladding oxide, with a 190 μm spacing between the two heating wires, all sharing a common ground plate. In our simulations, we applied electrical current solely to one micro-heater (MH1) to examine the effects of thermal crosstalk within the device. As depicted in Fig. S1(a), MH2, MH3, and MH4 remained at stable temperatures, while MH1 showed significant localized heating. Assuming ideal thermal conductivity at the phase shift points, the silicon nitride waveguide beneath inactive MHs remains unaffected. The heat from the active MH1 penetrates vertically through the SiO_2_ cladding, reaching directly beneath it with minimal lateral spread.

Fig. S1(b) illustrates how the phase shift amplitude at each of the four phase shift points responds to varying injection currents when only MH1 is active. At a current of 97 mA, PS1 undergoes a complete 1π phase shift, whereas PS1 experiences a minimal change of only 0.043π. Defining thermal crosstalk as the ratio of unintended phase change in neighboring waveguides to that in the directly heated waveguide, we observe a minimal crosstalk of 4.3% in our design. This low percentage indicates substantial thermal isolation provided by the MH arrangement, ensuring that the influence on phase shifters situated 190 µm apart is negligible.

Similarly, in Fig. S1(c), (e), and (g), when the same injection currents are independently applied to the other three MHs, we observe highly localized temperature increases at each respective MH, with stable max temperature variations within 3K, showcasing the setup's consistency and repeatability. Further analysis depicted in Fig. S2(d), (f), and (h) demonstrates that in all cases of individual MH modulation, thermal crosstalk remains below 10%. Notably, the highest interference occurred with MH4, influencing PS2 by 6.9%.

These findings confirm that phase shift noise due to thermal leakage between adjacent PSs is minimal, allowing each MH to independently modulate its corresponding phase shifter. This capability highlights the device's precision and effectiveness in applications requiring meticulous thermal management and phase control, such as in advanced photonic circuits where precise optical signal modulation is critical. This setup demonstrates our approach's effectiveness in integrating robust, efficient MHs into photonic devices, ensuring precise control over phase shifts while maintaining high performance and structural integrity


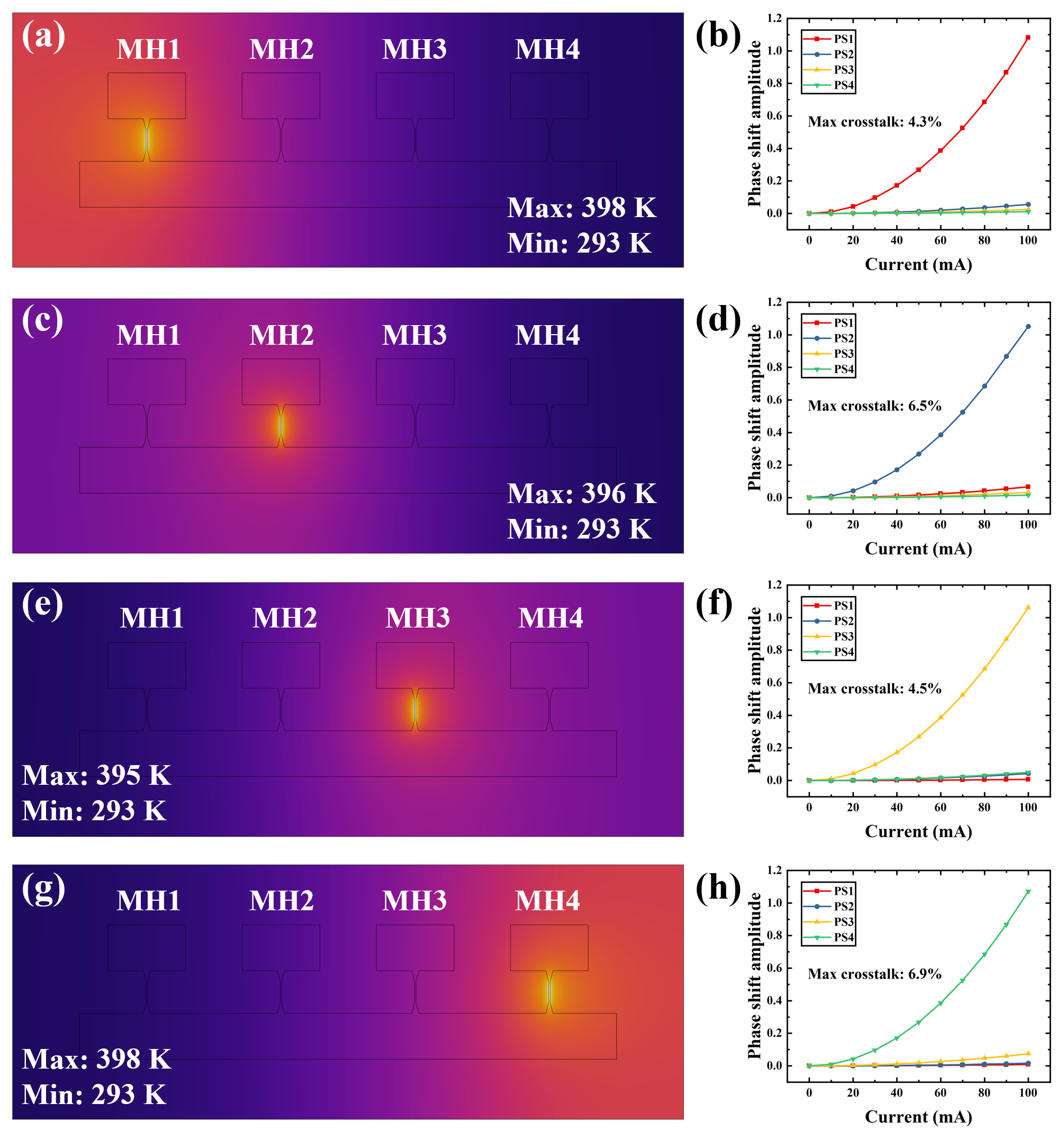


Fig. S1 Simulated temperature distribution when only (a) MH1, (c) MH2, (e) MH3, and (g) MH4 are operating, and the corresponding phase amplitude change of all PSs when the injection current is applied only to (b) MH1, (d) MH2, (f) MH3, and (h) MH4.

1. Fabrication process


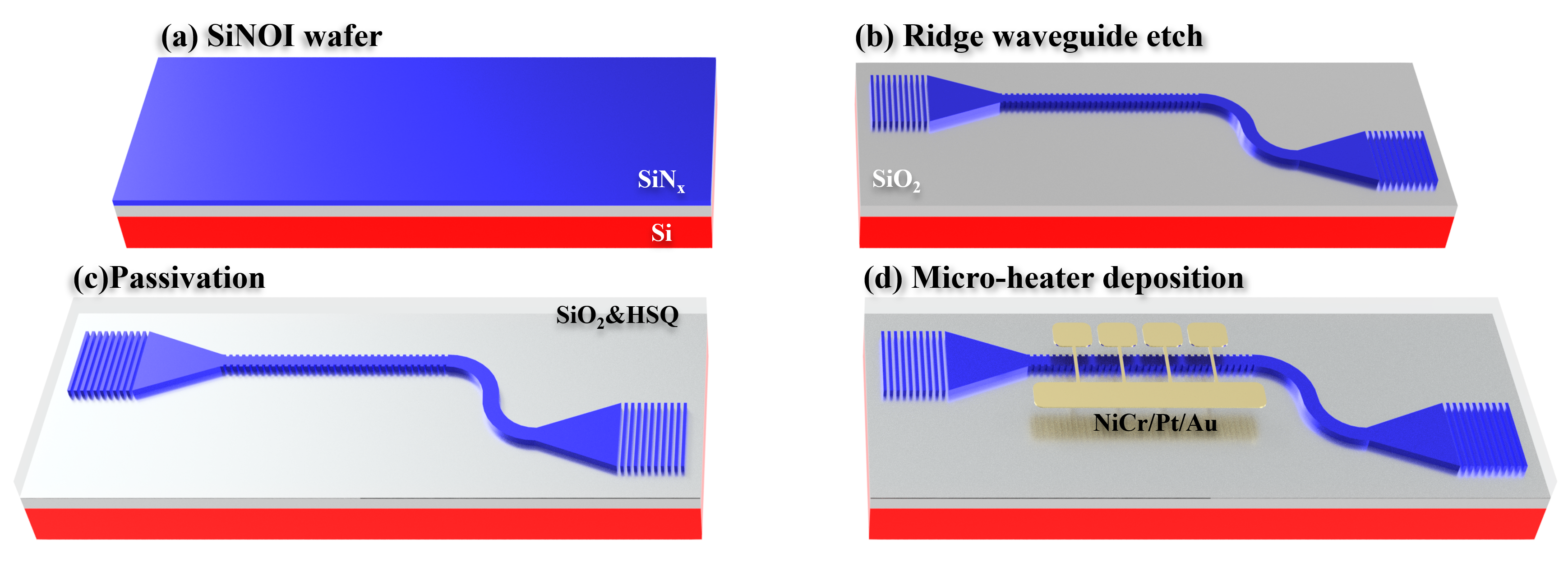


Fig. S2 Schematic of the PF fabricating process.

The fabrication process begins with a standard 3-inch silicon wafer of 500 µm thickness. First, thermal oxidation is performed to form a 5 µm thick oxide layer on the polished side of the wafer, serving as an isolation layer between the waveguide and the substrate. The wafer is then placed in an Oxford Instruments System 100 ICP 380 PECVD system, where a 340 nm thick SiNx film is deposited on the oxide layer at 300°C using a gas mixture of SiH₄:N₂ = 30 sccm:24 sccm, serving as the waveguide layer (see Fig. S2(a)). Subsequently, the wafer is diced into 11 mm × 12 mm square chips for further device fabrication.

The diced chips undergo ultrasonic cleaning in acetone, IPA, and RO water sequentially to ensure surface cleanliness. Before spin-coating, the chips are treated in a ultraviolet ozone cleaning system (UVOCS) for secondary surface cleaning and to enhance surface wettability, improving the adhesion of the photoresist to the waveguide layer. A 400 nm thick HSQ electron beam resist is then spin-coated onto the chips, followed by a 5-minute bake at 92°C on a hot plate to ensure complete solvent evaporation.

After baking, the chips are exposed using an EBPG 5200 EBL system with a 100 kV, 8 nA electron beam to pattern HSQ. The exposure includes grating couplers, waveguides, and sidewall gratings. The 3 nm minimum beam size ensures the accuracy of the grating structures. Following exposure, the chips undergo a sequential development process using 25% TMAH solution, RO water, and IPA. The IPA step enhances the verticality of the exposed HSQ sidewalls, improving pattern contrast.

The first dry etching process is then performed using an ICP SPTS Rapier DSiE system at room temperature with a gas mixture of 89 sccm C₄F₈ and 31 sccm SF₆, and a platen power of 28 W. The etching process is monitored by an interferometer, stopping when the remaining SiNx thickness reaches 10 nm. This thin residual layer protects the underlying oxide during the subsequent HSQ removal step. The chips are then rinsed in a 1:10 diluted HF solution for one minute. Since the etching rate of SiO₂ in HF is approximately 20 times faster than that of SiN, retaining the 10 nm SiN layer is essential to prevent the formation of suspended waveguides. Notably, to reduce random noise, a low molecular weight, high-contrast resist (HSQ) was used during grating patterning. Proximity effect correction (PEC) was applied to mitigate electron scattering in EBL. During ICP etching, a passivation gas (e.g., C₄F₈) was introduced to protect sidewalls from ion bombardment.

After the fabrication of the on-chip SiN structures (see Fig. S2(b)), planarization is required before MH fabrication. The chips are placed in an OIPT PECVD PlasmaPro 100 system, where a 400 nm SiO₂ film is deposited at 300°C using a gas mixture of 161 sccm Ar, 8.5 sccm SiH₄, and 710 sccm N₂O. A 600 nm thick HSQ layer is then spin-coated onto the chips, followed by annealing in a nitrogen-purged oven at 180°C (see Fig. S2(c)). Since the final MH thickness is only 230 nm, any surface roughness exceeding 100 nm could lead to circuit breakage at high temperatures, significantly reducing the MH lifespan and thermal efficiency. Surface profile measurements using a Stylus Profiler Dektak confirm that the cladding height variation above the 330 nm high waveguide is reduced to less than 50 nm.

After annealing, a double-layer PMMA resist is spin-coated onto the chips, consisting of 940 nm total thickness: AR-P 642 200k Anisole 15% PMMA and AR-P 679 950k Ethyl Lactate 2% PMMA, followed by baking at 180°C. The chips undergo a second round of EBL, and the MH patterns are developed using a 2.5:1 IPA: MIBK solution. The MH structures are fabricated using electron beam evaporation, sequentially depositing 60 nm NiCr, 120 nm Pt, and 50 nm Au onto the chip surface (see Fig. S2(d)). The chips are then immersed in acetone for a hot bath lift-off process. Finally, the samples undergo microscopic inspection to complete the fabrication process.

1. 50 GHz semiconductor mode-locked laser and superluminescent diode


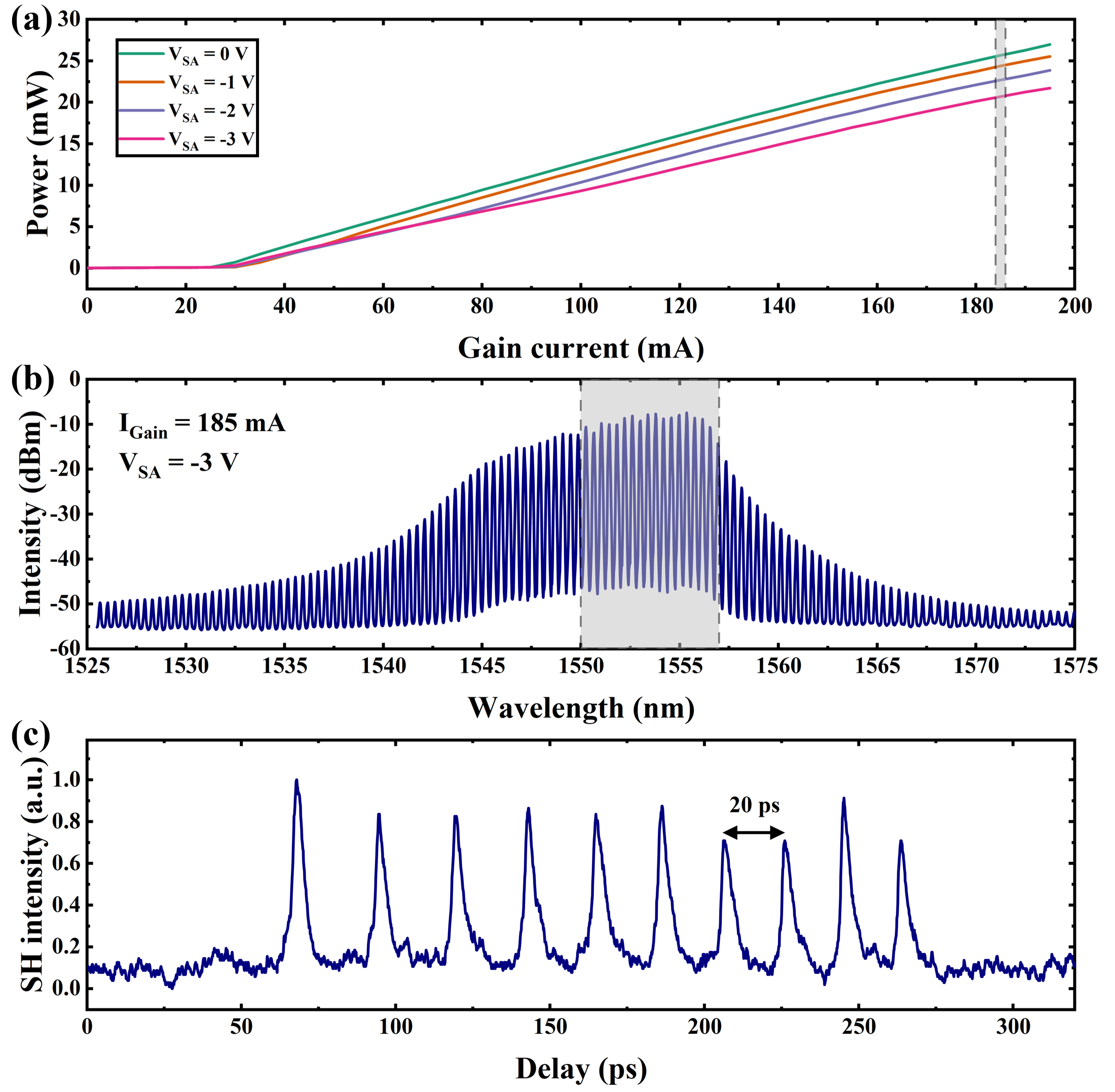


Fig. S3 (a) P-I curve for 𝐼*_gain_* ranging from 0 to 195 mA. (b) Optical spectrum at the gain section facet of the SMLL in the ML state, with a gain section injection current of 185 mA and a reverse bias voltage of -3 V applied to the SA section. (c) Full autocorrelation (AC) trace.

In our optical frequency division experiments, we employed a passive SMLL, constructed using the AlGaInAs/InP material system [4], to generate an OFC. This device is built with an asymmetric multi-quantum well (MQW) structure. A photograph of the SMLL, operating at a 50 GHz repetition frequency, is displayed in Fig. 4(b) of the manuscript, illustrating the device's optical microscope view.

The SMLL features a total cavity length of 890 μm, where the saturable absorber (SA) section is 15 μm long, and the gain section extends for 865 μm, separated by a 10 μm isolation groove. The ridge waveguide is 2.5 μm wide. As demonstrated in Fig. S3(a), with the SA section at 0 V, the device shows a threshold current of 25 mA and outputs 27 mW at a gain injection current of 195 mA. Passive mode-locking (ML) is achieved by applying a forward bias to the gain section while applying a reverse voltage to the SA section.

To evaluate the PF's capacity for optical frequency division, we adjusted the gain section injection current to 185 mA and applied a 3 V reverse bias to the SA section, prompting the device to exhibit free-running mode-locking with an average output power of 20 mW. The spectrum during this free-running period, shown in Fig. S3(b), centers at 1553 nm with a Full Width at Half Maximum (FWHM) of 6.3 nm. The SMLL's OFC, with a spacing of 50 GHz, is indicated by the FWHM of 4.5 nm. The gray section in Fig. S3(b) displays the reference signal generated by the input waveguide. The measured autocorrection (AC) trace, depicted in Fig. S3(c), shows a pulse separation of 20 ps, aligning precisely with the inverse of the 50 GHz ML frequency. The laser light signal, isolated by an isolator, is then coupled into the programmable PF.


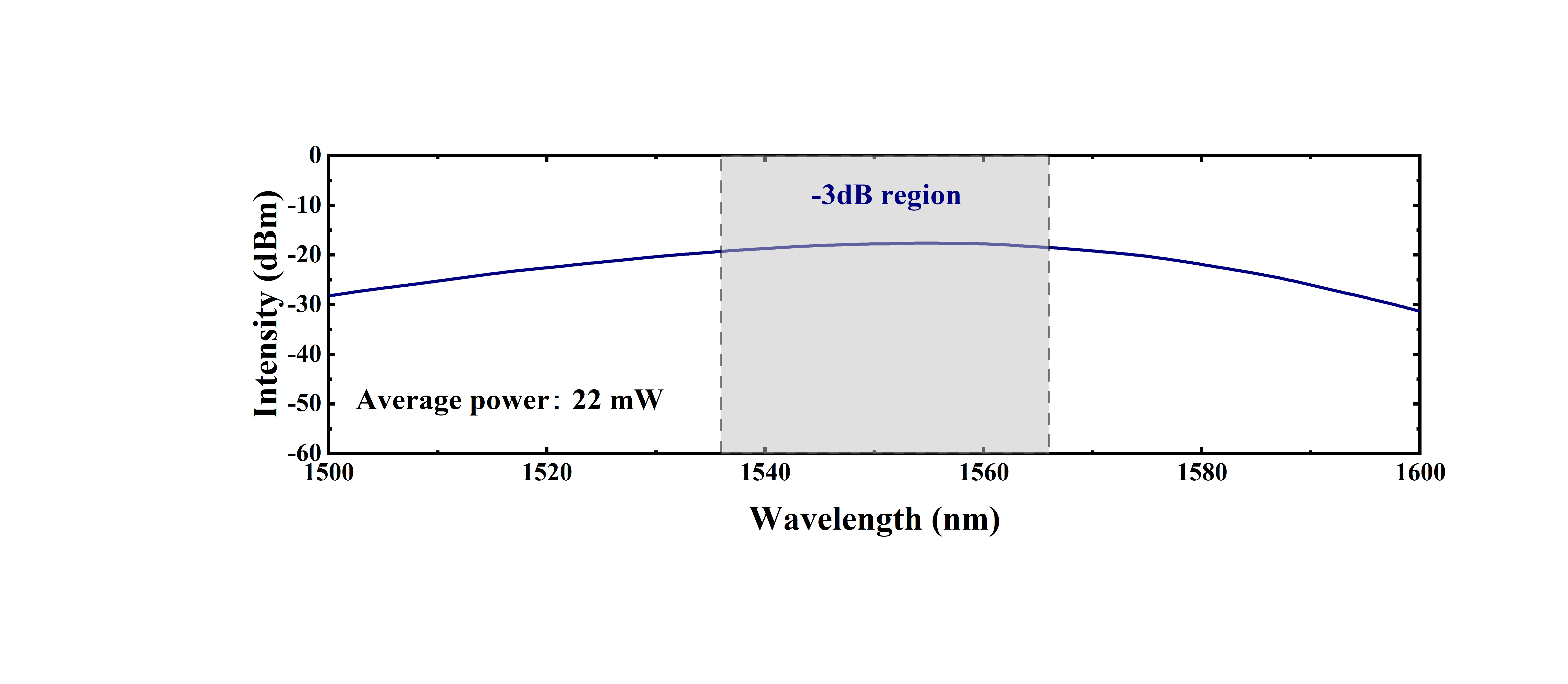


Fig. S4 Optical spectrum of the SLD at a drive current of 600 mA.

In our experiments to evaluate the PF filtering window performance, we used a benchtop fiber-coupled SLD light source from Thorlabs. This system integrates a super-radiant emitting diode, a control unit, and a temperature stabilization system (TEC) for precise operation. The SLD has a central wavelength of 1551 nm, an output power of 22 mW, and a -3 dB bandwidth of 30 nm, as shown in Fig. S4.

The SLD operates with an independent, high-precision low-noise constant current source and a dedicated temperature control unit, ensuring stable light output. An integrated microcontroller allows for detailed adjustment of the SLD’s optical power and temperature, in addition to monitoring for any system malfunctions. The device is built on an InP base, which contributes to its capabilities as a high-power, broadband light source. For the experiments, the output light from the SLD is coupled into subsequent optical systems through a SMF connected to a PC.

1. SiN_x_ on insulator grating coupler


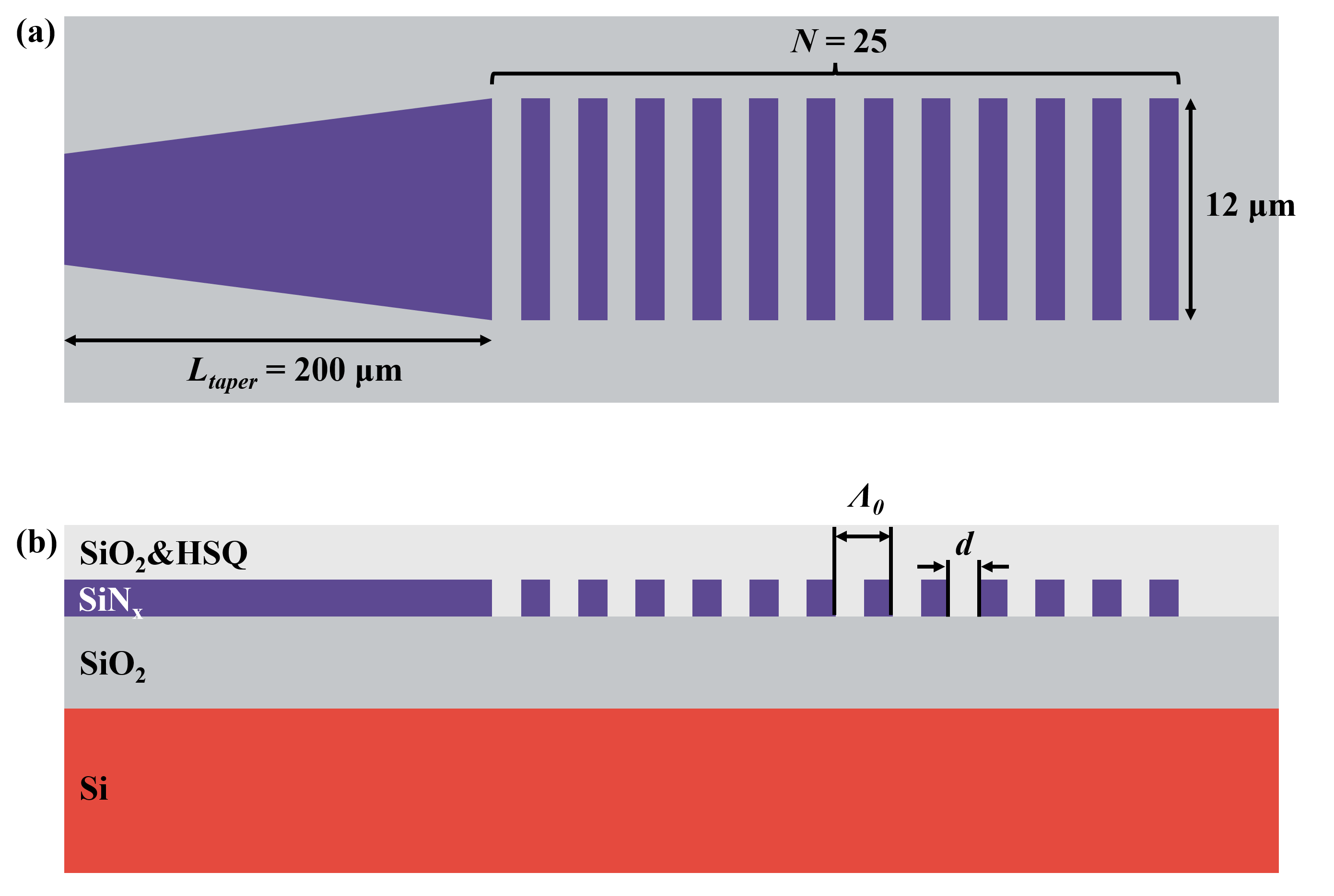


Fig. S5 Top view schematic (a) and side view schematic (b) of the SiN_x_ uniform grating coupler

Fiber couplers are key components in integrated photonics, widely used in wafer-level testing and packaging. SiN_x_-based couplers can achieve broader spectral bandwidth than Si-based couplers, but their coupling efficiency is somewhat limited due to their lower refractive index. Typical design strategies include: bottom reflectors [5], upward-enhancing gratings [6], and high refractive index overlay layers [7]. To balance manufacturability with high coupling efficiency, this study employs a conventional SiN_x_ uniform grating coupler, which only requires a single etching process and can be patterned for waveguides and grating couplers in a single electron beam lithography (EBL) exposure, simplifying the process and facilitating large-scale integration. Its structural schematic is shown in Fig. S5, featuring a 5 μm thick oxide isolation layer and a 340 nm thick SiN_x_ layer. The designed uniform grating parameters are as follows: a grating period (Ʌ_0_) of 1.19 μm and, a width of 12 µm, an etched region width (*d*) of 0.6 nm, and a grating fill factor of approximately 0.5. A single grating coupler consists of 25 grating periods. The coupling mode is primarily transverse electric, coupling out from the left side of the grating waveguide, with the majority of the electric field diffracting upward and aligning with the SMF mode. To enhance mode-matching efficiency, a 15° tilted vertical coupling scheme is employed. Additionally, in the experiments, the grating coupler is connected to an on-chip single-mode waveguide via a 200 μm long tapered waveguide, ensuring a smooth mode transition and minimizing end-face reflection losses. The fabricated grating coupler is shown in Fig. 3(d) of the manuscript.

Fig. S6 presents our simulated and experimental spectral graphs using an SLD as the input source. The green line in the figure is the spectral graph measured by connecting the SLD directly to the OSA via SMF, used as a reference signal. The orange line represents the simulated transmission spectra in Lumerical FDTD, where the SLD light source enters through the input grating coupler, propagates through a 600 μm long ridge waveguide, and is subsequently out-coupled into an SMF via the output grating coupler. At the wavelength of 1550 nm, the simulated coupling efficiency is -4.7 dB, not considering the effects of cladding reflection, fiber end-face reflection, and propagation losses. Subsequently, SiN_x_ ridge waveguides with identical parameters, along with their corresponding grating couplers, were fabricated using the aforementioned manufacturing process and experimentally characterized. As shown in the purple line in Fig. S6, at 1550 nm, the experimental output of the grating coupler showed an attenuation of 14 dB relative to the SLD reference signal. Further measurements indicated that the SiN_x_ ridge waveguide, after being covered with a 400 nm PECVD-deposited SiO_2_ cladding, had a propagation loss of 1.53 dB/cm. Subtracting the waveguide propagation losses, the actual coupling efficiency of a single grating coupler was calculated to be -6.95 dB. Furthermore, compared to the simulation results, the measured -3 dB bandwidth broadened to 55 nm, demonstrating robust broadband coupling characteristics.


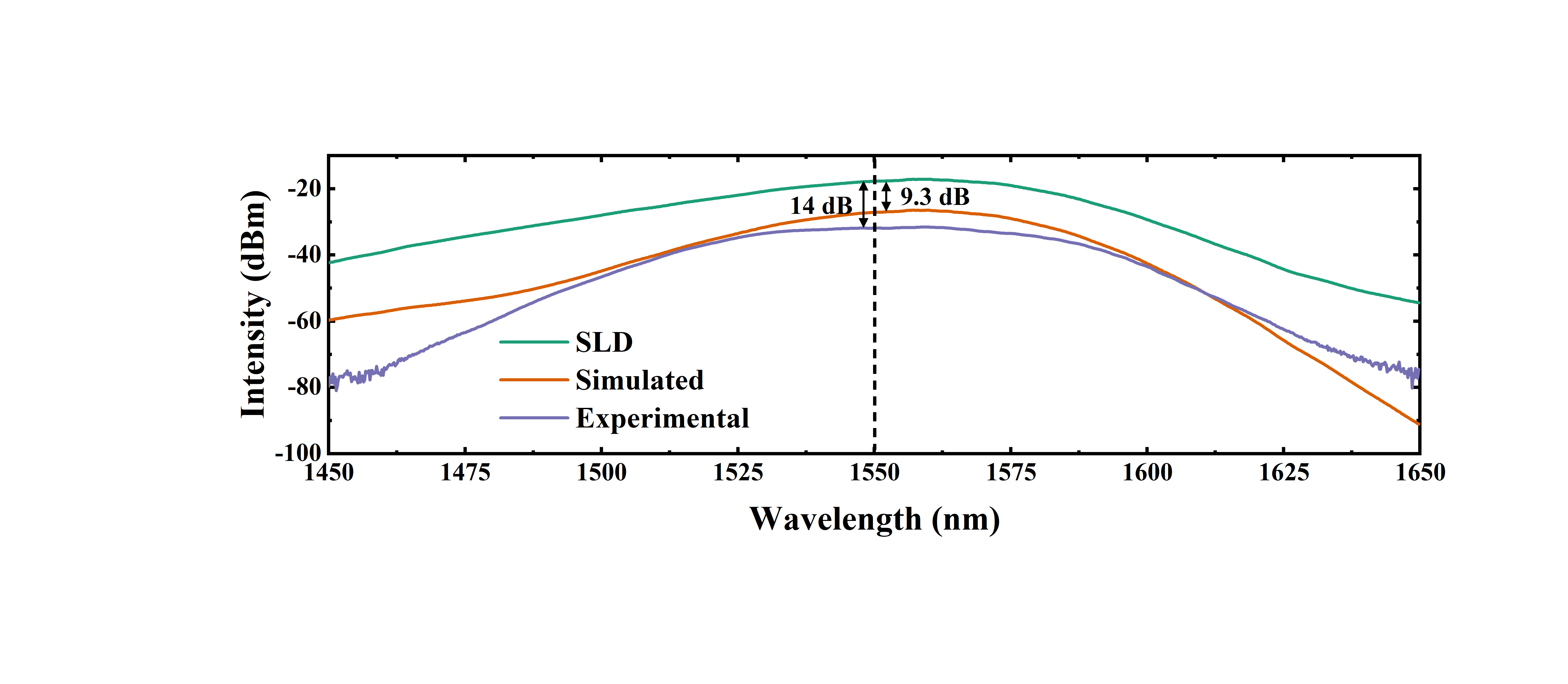


Fig. S6 Measured spectrum of the SLD (green), simulated transmission spectrum of a 600 μm ridge waveguide (orange), and experimentally obtained transmission spectrum of the same 600 μm ridge waveguide (purple).

References

1. P. Dong et al., “Low power and compact reconfigurable multiplexing devices based on silicon microring resonators,” Optics Express, vol. 18, no. 10, p. 9852, Apr. 2010, doi: 10.1364/oe.18.009852.
2. M. R. Watts, W. A. Zortman, D. C. Trotter, G. N. Nielson, D. L. Luck, and R. W. Young, “Adiabatic Resonant Microrings (ARMs) with Directly Integrated Thermal Microphotonics,” Conference on Lasers and Electro-Optics/International Quantum Electronics Conference, vol. 435, p. CPDB10, Jan. 2009, doi: 10.1364/cleo.2009.cpdb10.
3. G. Cocorullo, F. G. Della Corte, I. Rendina, and P. M. Sarro, “Thermo-optic effect exploitation in silicon microstructures,” Sensors and Actuators a Physical, vol. 71, no. 1–2, pp. 19–26, Nov. 1998, doi: 10.1016/s0924-4247(98)00168-x.
4. M. Al-Rubaiee, B. Yuan, Y. Fan, S. Zhu, J. Marsh, and L. Hou, “Ultrastable 10 GHz Mode-Locked laser on Semi-Insulating substrate through RF injection locking,” IEEE Photonics Technology Letters, vol. 36, no. 19, pp. 1189–1192, Aug. 2024, doi: 10.1109/lpt.2024.3451690.
5. H. Zhang et al., “Efficient silicon nitride grating coupler with distributed Bragg reflectors,” Optics Express, vol. 22, no. 18, p. 21800, Sep. 2014, doi: 10.1364/oe.22.021800.
6. Y. Chen, R. Halir, Í. Molina-Fernández, P. Cheben, and J.-J. He, “High-efficiency apodized-imaging chip-fiber grating coupler for silicon nitride waveguides,” Optics Letters, vol. 41, no. 21, p. 5059, Oct. 2016, doi: 10.1364/ol.41.005059.
7. X. Zhou and H. K. Tsang, “Optimized shift-pattern overlay for high coupling efficiency waveguide grating couplers,” Optics Letters, vol. 47, no. 15, p. 3968, Jul. 2022, doi: 10.1364/ol.464652.
